# Impact of cardiorespiratory fitness and diabetes status on cardiovascular disease and all-cause mortality: An NHANES retrospective cohort study

**DOI:** 10.1016/j.ahjo.2024.100395

**Published:** 2024-04-17

**Authors:** Gwendolyn A. Ung, Kevin H. Nguyen, Alvin Hui, Nathan D. Wong, Elizabeth H. Dineen

**Affiliations:** a Western University of Health Sciences College of Osteopathic Medicine of the Pacific-Pomona, CA, United States of America; b Division of Cardiology, University of California, Irvine School of Medicine, Irvine, CA, United States of America; c Department of Cardiovascular Diseases, Mayo Clinic, Jacksonville, FL, United States of America

**Keywords:** Cardiorespiratory fitness, Diabetes, Cardiovascular disease, Mortality

## Abstract

High cardiorespiratory fitness (CRF) is associated with decreased mortality in people with pre-diabetes (pre-DM) and diabetes mellitus (DM); however, the degree to which CRF attenuates the risk of cardiovascular disease (CVD)-related and all-cause mortality is unclear.

**Study objective:**

We examined the impact of CRF status on CVD-related morbidity and all-cause mortality in non-DM, Pre-DM, and DM populations.

**Design and setting:**

13,968 adults from the Third US National Health and Nutrition Examination Survey (NHANES III) were stratified into non-DM, pre-DM, or DM groups based on HbA1c levels. VO_2_Max was calculated using the Fitness Registry and Importance of Exercise: A National Database (FRIEND) equation.

**Participants:**

Participants were categorized into tertiles of VO_2_Max; first VO_2_Max tertile was the lowest VO_2_Max and third VO_2_Max tertile was the highest.

**Main outcome measure(s):**

Cox regression was used to analyze the relationship between glycemic levels, VO_2_Max, and CVD-related and all-cause mortality.

**Results:**

Those with DM in the highest fitness tertile had CVD (HR 0.13; 95 % CI 0.06, 0.27; *p* < 0.0001) and all cause (HR 0.28; 95 % CI 0.21, 0.38; p < 0.0001) mortality rates as low or lower than those with pre-DM (CVD HR 1.02; 95 % CI 0.78, 1.33 *p* < 0.892; all cause HR 0.96; 95 % CI 0.83, 1.12; *p* < 0.5496) or non-DM (CVD HR 0.65; 95 % CI 0.52, 0.80; *p* < 0.0001; all cause HR 0.61; 95 % CI 0.55, 0.68; p < 0.0001) at lower fitness levels. Regardless of DM status, there was lower all-cause mortality with higher CRF levels.

**Conclusions:**

Higher fitness levels in DM individuals are associated with total and CVD mortality rates as low or lower than those without DM with lower fitness.

## 1 Introduction

Cardiovascular disease (CVD) is the leading cause of death worldwide, accounting for approximately 17.8 million deaths and a total of 330 million years of life lost in 2017 [1]. Previous studies evaluating the impact of lifestyle modifications have found obesity to be a strong risk factor for both CVD and diabetes, demonstrating higher CVD and all-cause mortality in obese individuals with Type 2 diabetes mellitus (DM) and the general population [2,3]. Furthermore, physical inactivity and sedentary behavior are two risk factors that impact cardiorespiratory fitness (CRF), a strong predictor of CVD and all-cause mortality [4]. Few studies have compared both groups with pre-diabetes (pre-DM) and those with DM and stratified them based on physical fitness level using VO_2_Max, measured in milliliters of oxygen (O_2_) per kilogram of bodyweight per minute (mL/kg/min), as a method to assess the risk of CVD and all-cause mortality. Within a large sample of adults representative of the US population, we examined the level of physical fitness in relation to the incidence of CVD and total mortality according to the presence of pre-DM or DM. Of interest was whether high CRF attenuates the risk of cardiovascular-related and all-cause mortality in those without diabetes (non-DM), Pre-DM, and DM populations.

## 2 Methods

Data were extracted from the Third National Health and Examination Survey (NHANES III) 1988–1994, a national cross-sectional survey that serves to collect and analyze data from a nationally representative sample of non-institutionalized U.S. children and adults. As a comprehensive health-related survey, NHANES III includes data from physical examinations, physiological measurements, and laboratory findings on a total of 39,695 civilians aged 2 months and older. Of these individuals, there were 13,968 adult participants 20-<79 years of age selected for the study. The FRIEND table [5] provided data on individuals who were 20 years of age or older and < 79 years of age, and therefore this study was only able to perform VO_2_Max calculations on these participants. Participants without weight and BMI data were also excluded from the final study analysis. Details on NHANES data collection can be found elsewhere [6], however in brief, participants were interviewed at home by trained personnel and at a mobile examination center by a physician. Participants were provided with informed consent for both the home interview and mobile center examination. The current study utilized de-identified, publicly available data and was thus exempt from institutional review board review.

**Fig. 1:**
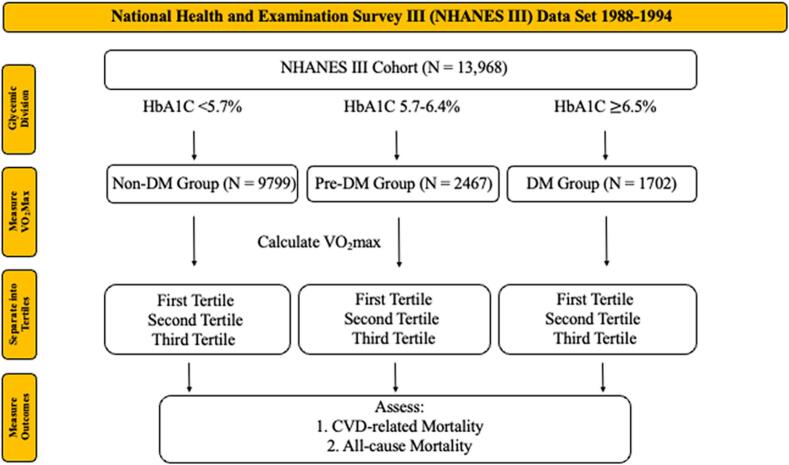
Experimental Design flow chart. Cardiovascular Disease (CVD), Diabetes Mellitus (DM), Hemoglobin A1C (HbA1C), National Health and Examination Survey III (NHANES III).

This study's primary outcomes of interest were all-cause and cardiovascular (CV) mortality within each glycemic group: non-DM, pre-DM, and DM. These groups were analyzed based on increasing levels of cardiorespiratory fitness determined by one's VO_2_Max. Cardiovascular death was determined using the specific ICD-10 cardiovascular code from the NHANES database based on National Death Index linkage through December 31, 2018. Fig. 1, displays the flow chart depicting the breakdown of the NHANES III cohort and into its subsequent groups for analysis and measure outcomes.

Baseline characteristics were described as mean ± standard deviations or proportions according to DM status (non-DM, pre-DM, and DM) and VO_2_Max tertiles (1st, 2nd, and 3rd VO_2_Max tertiles). A log-rank test and Kaplan-Meier plot were used to establish unadjusted associations between VO_2_Max tertiles and all-cause and CV mortality. The relationship between CRF and all-cause and CV mortality was determined using Cox proportional hazard models. Selected participants were separated into one of three glycemic groups based on their hemoglobin A1c (HbA1c) measurements: non-DM, Pre-DM, or DM. We utilized the American Diabetes Association criteria to determine the HbA1c ranges 5.7–6.4 % and ≥ 6.5 % for Pre-DM and DM participants, respectively.

Each participant's VO_2_Max (mL / kg / min) was calculated using the Fitness Registry and the Importance of Exercise: A National Database (FRIEND) equation^6^: 79.9 - (0.39 x age) - (13.7 x gender [0 = male; 1 = female]) - (0.127 x weight [lbs]). Then, within each DM status group, the participants were further categorized into three tertiles based on their VO_2_Max. Participants within tertile 1 have VO_2_Max ≤ 30.9, tertile 2 have VO_2_Max > 30.9- ≤39.3 mL*kg/min and tertile 3 VO_2_max > 39.3. Accordingly, the relationship between glycemic levels, VO_2_Max, and CVD-related mortality in each of the three VO_2_Max tertiles was analyzed using multivariate, cox regression hazard analysis, with the first VO_2_Max tertile serving as the reference group.

We used two models in our analysis comparing each glycemic group stratified by VO_2_Max tertiles in relation to: (1) an unadjusted model: the mortality outcome and (2) a fully adjusted model: systolic blood pressure (SBP), diastolic blood pressure (DBP), hypertension status, hypertension medication, total cholesterol, cholesterol medication, tobacco status, diabetes medication and insulin. Age, sex, and BMI were not included in our adjusted model due to the FRIEND equation already accounting for these variables. The fully adjusted models are our primary models for each glycemic group. A *p*-value of <0.05 was used to define statistical significance. All analyses performed utilized SAS 9.4 (SAS Institute, Cary, North Carolina).

## 3 Results

Of the total 13,968 individuals (51 % female), 9799 were in the non-DM group, 2467 were in the Pre-DM group, and 1702 were in the DM group. Those with pre-DM or DM (compared to non-DM) were more likely to be older, less likely to be female, more likely to be black, and more frequently to have higher total cholesterol, blood pressure, and weight (Table 1a). Individuals with higher VO_2_Max tertiles (compared to the first tertile) tend to be younger, less likely to be female, more likely to be Mexican-American and have lower cholesterol, blood pressure, and weight (Table 1b). Mean follow-up time was 17.5 years overall (18.4 for non-DM, 16.1 for pre-DM, and 14.4 for DM).

**Table 1a:** Baseline Characteristics for Diabetes Status.

Variables	Total	Diabetes status (DM)			
		Non-DM	Pre-DM	DM	p-value
N (%)	13,968	9799	2467	1702	< 0.001
Age (± SD) (Years)	45 (± 17)	42 (± 16)	55 (± 17)	55 (± 17)	< 0.001
Female (%)	51	53	44	49	< 0.001
Race ethnicity (%)					
Non-Hispanic White	39	43	31	30	< 0.001
Non-Hispanic Black	28	24	40	36	< 0.001
Mexican-American	29	29	24	31	< 0.001
Other	4	4	5	4	< 0.001
Laboratory values					
Systolic Blood Pressure (mmHg)	125 (± 34)	122 (± 33)	131 (± 32)	135 (± 44)	< 0.001
Diastolic Blood Pressure (mmHg)	75 (± 34)	75 (± 33)	77 (± 34)	78 (± 42)	< 0.001
BMI (kg/m^2^)	26 (± 4)	25 (± 6)	27 (± 6)	27 (± 6)	< 0.001
Weight (lbs)	159 (± 31)	157 (± 30)	166 (± 31)	164 (± 34)	< 0.001
Total Cholesterol (mg/dL)	204 (± 45)	199 (± 42)	218 (± 44)	211 (± 55)	< 0.001
HDL-Cholesterol (mg/dL)	52 (± 16)	53 (± 16)	50 (± 15)	47 (± 15)	< 0.001
Questionnaire data					
Taking insulin or diabetes medication (%)	5	0	0	42	< 0.001
Cholesterol Medication (%)	3	2	5	6	< 0.001
Ever told you have hypertension (%)	3	2	5	9	< 0.001
Do you smoke cigarettes now? (%)	29	28	34	24	< 0.001
Now taking prescribed medicine for hypertension (%)	13	9	20	29	< 0.001

Abbreviations: SD, standard deviation. BMI, body mass index, HDL, high density lipoprotein.

**Table 1b:** Baseline characteristics for VO_2_Max tertiles.

Variables	Total	VO_2_Max tertiles			
		First tertile	Second tertile	Third tertile	p-value
N(%)	13,968	4656	4656	4656	<0.001
Age (± SD) (years)	45(±17)	61(±13)	44(±15)	21(±9)	<0.001
Female (%)	51	51	35	14	<0.001
Race ethnicity (%)					
Non-Hispanic White	39	43	33	25	<0.001
Non-Hispanic Black	28	30	35	35	<0.001
Mexican-American	29	24	33	43	<0.001
Other	4	30	32	38	<0.001
Laboratory values					
Systolic Blood Pressure (mmHg)	125(±34)	134(±34)	122(±39)	118(±28)	<0.001
Diastolic Blood Pressure (mmHg)	75(±34)	77(±33)	76(±37)	74(±31)	<0.001
BMI (kg/m^2^)	26(±4)	28(±6)	26(±5)	24(±5)	<0.001
Weight (lbs)	159(±31)	165(±32)	159(±31)	154(±30)	<0.001
Cholesterol (mg/dL)	204(±45)	221(±45)	203(±43)	190(±40)	<0.001
HDL-Cholesterol (mg/dL)	52(±16)	53(±17)	51(±16)	51(±15)	<0.001
Questionnaire data					
Taking insulin or diabetes medication (%)	5	10	4	1	<0.001
Cholesterol medication (%)	3	6	2	<1	<0.001
Ever told you have hypertension (%)	3	7	3	<1	<0.001
Do you smoke cigarettes now? (%)	29	20	29	37	<0.001
Now taking prescribed medicine for hypertension (%)	13	28	10	2	<0.001

Abbreviations: SD, standard deviation.

For all-cause mortality, compared to those in the lowest VO_2_Max tertile, adjusted hazard ratios (HRs) for those in VO_2_Max tertile 2 were 0.61 (95 % CI, 0.55 to 0.68) and 0.63 (95 % CI, 0.54 to 0.75), for those with without DM and DM, respectively (both *p* < 0.001) (Table 2). All three groups (non-DM (p < 0.001), Pre-DM (*p* < 0.0001), and DM (p < 0.0001)) within VO_2_Max tertile 3 showed significantly decreased risk of all-cause mortality. The same three groups, (non-DM (p < 0.001), Pre-DM (p < 0.0001), and DM (p < 0.0001)), within VO_2_Max tertile 3 had an adjusted HR of all-cause mortality of 0.29 (95 % CI, 0.25 to 0.24), 0.28 (95 % CI, 0.22 to 0.37), 0.28 (95 % CI, 0.21 to 0.38), respectively. Nevertheless, the second VO_2_Max tertile of pre-DM was not associated with all-cause mortality compared to non-DM and DM (*p* = 0.5496) with a HR of 0.96 (95 % CI, 0.83 to 1.12).

**Table 2:** Hazard ratios (HR), All-cause mortality, and VO_2_Max tertiles.

HbA1c (glycated hemoglobin %)	VO_2_max tertile 1	VO_2_max tertile 2 HR (95 % CI)	p-value	VO_2_max tertile 3 HR (95 % CI)	p-value
<5.7 (non-DM)	REF	0.61 (0.55, 0.68)	<0.0001	0.29 (0.25, 0.34)	<0.001
5.7 ≤ HbA1c < 6.5 (pre-DM)	REF	0.96 (0.83, 1.12)	0.5496	0.28 (0.22, 0.37)	<0.0001
≥6.5 (DM)	REF	0.63 (0.54, 0.75)	<0.0001	0.28 (0.21, 0.38)	<0.0001

Adjusted for: Systolic blood pressure (SBP), Diastolic blood pressure (DBP), total cholesterol, HDL, hypertension medications, cholesterol medications, tobacco use, ethnicity. Abbreviations: HR, hazard ratio, DM, diabetes mellitus, CI confidence interval, SBP, systolic blood pressure, DBP, diastolic blood pressure, HDL high density lipoprotein.

**Fig. 2:**
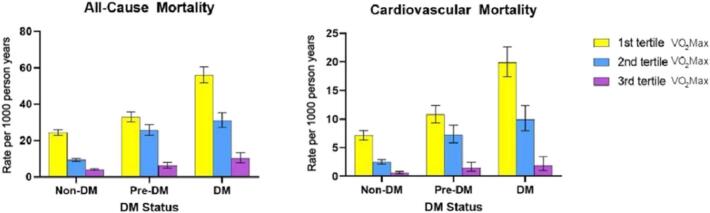
Mortality rates across VO_2_Max tertiles by DM status. Diabetes Mellitus (DM).

When comparing the risk of CV-related mortality, the non-DM (*p* < 0.0001) and DM (*p* < 0.004) group showed significantly lower risk compared to the control group in both VO_2_Max tertile 2 and 3. Within the highest VO_2_Max tertile, there was a significant reduction in CV mortality in all glycemic statuses compared to the reference group (*p* < 0.0001). Within VO_2_Max tertile 3, the non-DM group (p < 0.0001), the pre-DM group (< 0.0001), and DM group had an adjusted HR of 0.20 (95 % CI, 0.15 to 0.28), 0.33 (95 % CI, 0.2 to 0.56), and 0.13 (95 % CI, 0.06 to 0.27), respectively. This is in contrast to the non-DM and DM groups with adjusted hazard ratios of 0.65 (95 % CI, 0.52 to 0.80) and 0.65 (95 % CI, 0.49 to 0.87), respectively, within VO_2_Max tertile 2. Thus, no tertile-wide statistically significant differences were observed within the VO_2_Max tertiles when comparing non-DM and pre-DM to DM for all-cause and CV mortality. In summary, Fig. 2 depicts individuals with higher CRF status, reflected in higher tertiles of VO_2_Max, are associated with decreasing rates of all-cause and cardiovascular mortality. Table 2, Table 3 summarize the adjusted hazard ratios between glycemic status and VO_2_Max tertiles.

**Table 3:** Hazard ratio (HR), CV mortality, and VO_2_Max tertile.

HbA1c (glycated hemoglobin %)	VO_2_max tertile 1	VO_2_max tertile 2 HR (95 % CI)	p-value	VO_2_max tertile 3 HR (95 % CI)	p-value
< 5.7 (non-DM)	REF	0.65 (0.52, 0.80)	<0.0001	0.20 (0.15, 0.28)	<0.0001
5.7 ≤ HbA1c < 6.5 (pre-DM)	REF	1.02 (0.78, 1.33)	0.892	0.33 (0.20, 0.56)	<0.0001
≥6.5 (DM)	REF	0.65 (0.49, 0.87)	<0.004	0.13 (0.06, 0.27)	<0.0001

Adjusted for: SBP, DBP, total cholesterol, HDL-C, hypertension medication, cholesterol medication, tobacco use, ethnicity. Abbreviations: HR, hazard ratio, DM, diabetes mellitus, CI confidence interval, SBP, systolic blood pressure, DBP, diastolic blood pressure, HDL high density lipoprotein.

## 4 Discussion

Individuals in the highest VO_2_Max tertile, regardless of glycemic status, demonstrated a statistically significant lower risk of both all-cause and CV related mortality. Of note, those with DM in the highest fitness VO_2_Max tertile had CVD (HR 0.13; 95 % CI 0.06, 0.27) and total (HR 0.28; 95 % CI 0.21, 0.38) mortality rates that were as low or lower than those with pre-DM or normoglycemic at lower fitness levels. Our study is unique in examining the relation of estimated V0_2_Max with CV and total mortality according to glycemic status and is among the first to apply the FRIEND equation to a large diverse sample representative of the US population. It demonstrates the utility of this equation as a way to easily measure VO_2_Max and assess a patient's physical health and risk factors for developing a cardiovascular-related mortality event.

The past three decades have demonstrated a plethora of evidence establishing the strong association between optimal cardiorespiratory fitness (CRF) and decreased risk of cardiovascular disease (CVD) and all-cause mortality [4,7,8]. Our results help to support these data. In fact, CRF has been named as a major prognostic marker and the fourth‑leading risk factor for CVD [9]. In addition to many review articles around the world having confirmed the inverse relationship between highly physically active individuals and lower rates of CVD, there have also been randomized clinical trials that support a causal relationship [10]. These randomized trials have found that compared to the least active individuals, the most active individuals roughly have a 30 % to 40 % risk reduction of CVD and CHD [10,11].

Not only are our findings consistent with these results mentioned above, but they are also in line with other papers highlighting the amount of risk reduction in those who partake in moderate or high intensity physical activity. In the 2008 Physical Activity Guidelines, the panel conducting the systematic review concluded that “greater amounts of activity appear to provide greater benefit” [11]. This review found results similar to our study where individuals with moderate physical activity compared to low physical activity had a 20 % to 25 % reduced risk of CVD and those with high physical activity compared to low had a 30 % to 35 % risk reduction [11]. While the amount of risk reduction differs, our study confirms the pattern seen with other studies where an increased CRF level and physical activity leads to decreased risk of all-cause and CVD mortality.

When considering the relationship between physical activity and CVD and all-cause mortality in non-DM, pre-DM, and DM individuals, there is limited data. In a study published in 2000, 1263 men with type 2 diabetes were followed over an average of 12 years to evaluate the association between low CRF and physical inactivity with mortality [12,13]. This study found a strong association with adjusted relative risk of 2.1 for death for those with lower fitness levels [12]. A similar study compared physical activity of 5125 diabetic female nurses and the incidence of CVD events and concluded a significant negative association with between physical activity and CVD risk [14]. However, few studies examine this relationship between CRF and mortality in diabetic populations, and those that do study a limited population [[11], [12], [13], [14], [15]], suggesting the need for more research on the topic.

Our results suggest that regardless of glycemic status, a higher VO_2_Max, and therefore CRF, is associated with a lower risks for both CVD and all-cause mortality. These results suggest that CRF can be an additional tool for physicians in routine clinical practice to assess CVD risk especially since low CRF has been found to be associated with a 2- to 5-fold increase in CVD or all-cause mortality [5]. Utilizing the FRIEND equation can serve as an accessible marker to quickly assess a patient's CRF in the office without the need for a formal fitness test. Overall, utilizing CRF to stratify risk offers health professionals an additional opportunity to improve patient care and encourage lifestyle changes to reduce CVD and all-cause mortality [4,16,17]. Our study is one of few to compare the CVD and all-cause mortality among non-DM, pre-DM, and DM patients. It provides unique data to suggest that improving CRF will decrease CVD and all-cause mortality in DM patients.

Several limitations within this study may exist that hinder the generalizability of the data. There are several reasons that the HR was low within our data. This is an observational study that uses an already completed data such as NHANES, therefore, casual relationships cannot be established. Furthermore, due to the study's observational design, we cannot rule out further confounding due to both unmeasured and measured factors related both mortality and VO2Max, including age, sex, and weight which are part of the FRIEND equation. Another limitation of this study is that we did not analyze men and women independently. A second limitation involves the usage of an estimated VO_2_Max as opposed to calculating VO_2_Max with a fitness test directly. The FRIEND equation was used as it was found to closely parallel the measured VO_2_Max values with a 100.4 % percent predicted value [16]. This can be attributed to the larger and more diverse sample size used to derive the eq. (7759 subjects: 4601 men, 3158 women, mean age 45.9 ± 12.8 years) from ten laboratories across the US contributing data, as opposed to other equations that did not include such diversity [16]. Using the FRIEND equation allowed us to conduct a retrospective study on a large database such as NHANES III.

Future studies can include larger patient populations with more pre-DM and DM individuals and with greater racial/ethnic diversity (including Asian populations) for comparison. Furthermore, stratification of men and women would allow for the detection of VO_2_Max differences based on sex. While this study contained a large study population, the number of non-DM compared to the pre-DM and DM groups was significantly larger and could possibly explain the consistent statistically significant results. More research into why the pre-DM individuals in VO_2_Max tertile 2 had a lower HR in both CVD and all-cause mortality is needed before drawing a full conclusion in this patient group. With an increasing number of individuals developing pre-DM and DM, more research to understand the relationship between CRF and mortality in this population is needed.

In conclusion, we show higher levels of CRF to be associated with lower rates of total and CVD mortality regardless of glycemic status. Of note, those with DM in the highest fitness VO_2_Max tertile had CVD and total mortality rates that were as low or lower than those with pre-DM or normoglycemic at lower fitness levels, suggesting that higher fitness levels may help attenuate much of the excess mortality in those with DM.

## Funding

None of the authors have conflicts to report. No funding was used for this analysis or the preparation of this manuscript. The National Health and Nutrition Examination survey is funded by the Centers for Disease Control.

## CRediT authorship contribution statement

**Gwendolyn A. Ung:** Writing – review & editing, Writing – original draft, Validation, Methodology. **Kevin H. Nguyen:** Writing – review & editing, Writing – original draft, Validation, Methodology. **Alvin Hui:** Writing – review & editing, Writing – original draft, Validation, Methodology. **Nathan D. Wong:** Writing – review & editing, Supervision, Project administration, Methodology, Investigation, Conceptualization. **Elizabeth H. Dineen:** Writing – review & editing, Supervision, Project administration, Methodology, Conceptualization.

## Declaration of competing interest

The authors declare that they have no known competing financial interests or personal relationships that could have appeared to influence the work reported in this paper.

## References

[1] World Health Organization. Cardiovascular diseases (CVDs). Accessed July 11, 2023. https://www.who.int/news-room/fact-sheets/detail/cardiovascular-diseases-(cvds).

[2] Carbone S., Del Buono M.G., Ozemek C., Lavie C.J. (2019). Obesity, risk of diabetes and role of physical activity, exercise training and cardiorespiratory fitness. Prog. Cardiovasc. Dis..

[3] Lavie C.J., Ozemek C., Carbone S., Katzmarzyk P.T., Blair S.N. (2019). Sedentary behavior, exercise, and cardiovascular health. Circ. Res..

[4] Ross R, Blair SN, Arena R, Church TS, Després JP, Franklin BA, Haskell WL, Kaminsky LA, Levine BD, Lavie CJ, Myers J, Niebauer J, Sallis R, Sawada SS, Sui X, Wisløff U; American Heart Association Physical Activity Committee of the Council on Lifestyle and Cardiometabolic Health; Council on Clinical Cardiology; Council on Epidemiology and Prevention; Council on Cardiovascular and Stroke Nursing; Council on Functional Genomics and Translational Biology; Stroke Council. Importance of assessing cardiorespiratory fitness in clinical practice: a case for fitness as a clinical vital sign: a scientific statement from the American Heart Association. *Circulation*. 2016;134(24):e653-e699. doi:10.1161/CIR.0000000000000461. 27881567

[5] Kaminsky L.A., Imboden M.T., Arena R., Myers J. (2017). Reference standards for cardiorespiratory fitness measured with cardiopulmonary exercise testing using cycle ergometry: data from the Fitness Registry and the Importance of Exercise National Database (FRIEND) registry. Mayo Clin. Proc..

[6] Plan and operation of the Third National Health and Nutrition Examination Survey, 1988–94. Series 1: programs and collection procedures. *Vital Health Stat. 1*. 1994;(32):1–407. 7975354

[7] Sui X., LaMonte M.J., Blair S.N. (2007). Cardiorespiratory fitness as a predictor of nonfatal cardiovascular events in asymptomatic women and men. Am. J. Epidemiol..

[8] Blair SN, Kohl Iii HW, Paffenbarger RS, Clark DG, Cooper KH, Gibbons LW. Physical Fitness and All-cause Mortality A Prospective Study of Healthy Men and Women. https://jamanetwork.com/. 10.1001/jama.262.17.2395 2795824

[9] Fletcher G.F., Balady G., Blair S.N., Blumenthal J., Caspersen C., Chaitman B., Epstein S., Sivarajan Froelicher E.S., Froelicher V.F., Pina I.L., Pollock M.L. (1996 Aug 15). Statement on exercise: benefits and recommendations for physical activity programs for all Americans. A statement for health professionals by the Committee on Exercise and Cardiac Rehabilitation of the Council on Clinical Cardiology, American Heart Association. Circulation.

[10] Shiroma E.J., Lee I.M. (2010). Physical activity and cardiovascular health: lessons learned from epidemiological studies across age, gender, and race/ethnicity. Circulation.

[11] Physical Activity Guidelines Advisory Committee report, 2018. *To the Secretary of Health and Human Services. Part A: executive summary.*2018:A1-A7. https://health.gov/sites/default/files/2019-09/PAG_Advisory_Committee_Report.pdf. 10.1111/j.1753-4887.2008.00136.x 19178654

[12] Wei M., Gibbons L.W., Kampert J.B., Nichaman M.Z., Blair S.N. (2000). Low cardiorespiratory fitness and physical inactivity as predictors of mortality in men with type 2 diabetes. Ann. Intern. Med..

[13] Kokkinos P., Myers J. (2010). Exercise and physical activity: clinical outcomes and applications. Circulation.

[14] Hu F.B., Stampfer M.J., Solomon C., Liu S., Colditz G.A., Speizer F.E., Willett W.C., Manson J.E. (2001 Jan 16). Physical activity and risk for cardiovascular events in diabetic women. Ann. Intern. Med..

[15] Church T.S., LaMonte M.J., Barlow C.E., Blair S.N. (2005 Oct 10). Cardiorespiratory fitness and body mass index as predictors of cardiovascular disease mortality among men with diabetes. Arch. Intern. Med..

[16] Kaminsky L.A., Imboden M.T., Arena R., Myers J. (2017). Reference standards for cardiorespiratory fitness measured with cardiopulmonary exercise testing using cycle Ergometry: data from the fitness registry and the importance of exercise National Database (FRIEND) registry. Mayo Clin. Proc..

[17] Chul Lee D., Artero E.G., Sui X., Blair S.N. (2010). Mortality trends in the general population: the importance of cardiorespiratory fitness. J. Psychopharmacol..

